# Application Effectiveness of Segment IV Portal Vein Reconstruction for Early Postoperative Liver Function Recovery in Split Liver Transplantation

**DOI:** 10.3389/ti.2023.10808

**Published:** 2023-04-26

**Authors:** Imran Muhammad, Faisal U. L. Rehman, Feng Wang, Xiaopeng Xiong, Zhang Lianghao, Cai Jinzhen

**Affiliations:** ^1^ Organ Transplantation Center, The Affiliated Hospital of Qingdao University, Qingdao, China; ^2^ Precision Medicine Center of Oncology, The Affiliated Hospital of Qingdao University, Qingdao University, Qingdao, China

**Keywords:** liver transplantation, donors, split, liver function, portal reconstruction

## Abstract

The objective of this study was to investigate the significance of portal vein reconstruction in segment IV of the liver on early postoperative liver function recovery in split liver transplantation. The clinical data of patients of right trilobe split liver transplantation in our center were analyzed and divided into two groups, including a group without portal vein reconstruction and a group with portal vein reconstruction. Clinical data of alanine aminotransferase (ALT), aspartate transaminase (AST), albumin (ALB), creatinine (Cr), total bilirubin (TB), alkaline phosphatase (ALP), gamma-glutamyl Transferase (GGT), lactic acid (Lac), and international normalized ratio (INR) levels were analyzed. The technique of segment IV portal vein reconstruction is beneficial to the early postoperative recovery of liver function. Statistically, there was no significant effect of portal vein reconstruction in the IV segment of the liver on the recovery of liver function within 1 week after split liver transplantation. There was no significant difference in survival rate between the control group and reconstruction group over the 6 months follow-up period after surgery.

## Graphical Abstract

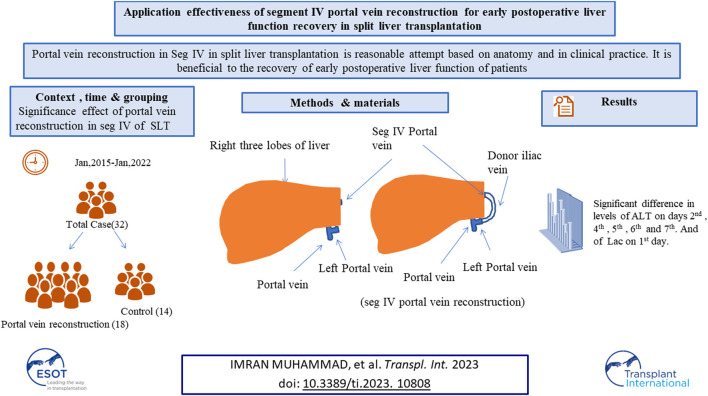

## Introduction

Since the first liver transplant was performed, with the continuous advancement of surgical techniques, extensive development, and clinical application of various new immunosuppressants, liver transplantation has become the most effective means of treating various end-stage liver diseases (1). Successful transplantation of a reduced volume liver to children, and right/left hemi-split liver transplantation performed on two adults have also been documented in the literature (2, 3). Expanding the source of donor livers has always been a major problem to be solved. According to statistics, the development of split liver transplantation can increase the number of donor livers, so it has become an important way for experts in the field of liver transplantation to solve the shortage of donor livers (4). In split liver transplantation, one donor liver is transplanted to two recipients, thereby expanding the source of donor livers.

There is no statistically significant difference in the graft and recipient survival rates at 1 year for those who have had whole liver transplantation and those who have undergone adult split liver transplantation (5, 6). In experienced transplantation institutions, split liver transplantation has a similar impact to whole liver transplantation, and its survival rate is comparable (7–9). The selection of donors and recipients is critical to the success of split liver transplantation. The ideal donor for splitting is someone who is young, has normal liver enzymes, hemodynamically stable, has no history of liver illness, and has a brief hospital stay (10, 11). Different donor splitting criteria have been suggested in previous studies, and they differ across nations and transplant institutions (12, 13).

A team disclosed two separate *in situ* split techniques for the fabrication of split grafts acceptable for two adult patients. In order to enhance the arterial supply to segment IV, they retain the common portal vein and the common hepatic duct with the right graft and the celiac axis with the left graft (14, 15). Another group released an evaluation, this time they described distinct anatomic situations following dissection of the portal subdivisions to segment IV, exposing the left hilar plate beneath the left portal vein, and surgery of the biliary ducts from segments II and III for traditional split liver transplantation (16).

In our center, the donor iliac blood vessels are used to bridge the partial segment IV portal vein branches that have been severed, thereby preserving the portal vein blood supply of the segment IV liver, ensuring functional liver volume, and improving the transplant rate of split liver transplantation. Our center’s exploration of portal vein reconstruction in segment IV liver for split liver transplantation is a reasonable attempt based on anatomy, and it is beneficial in clinical practice to the recovery of early postoperative liver function of patients.

## Patients and Methods

**FIGURE 1:**
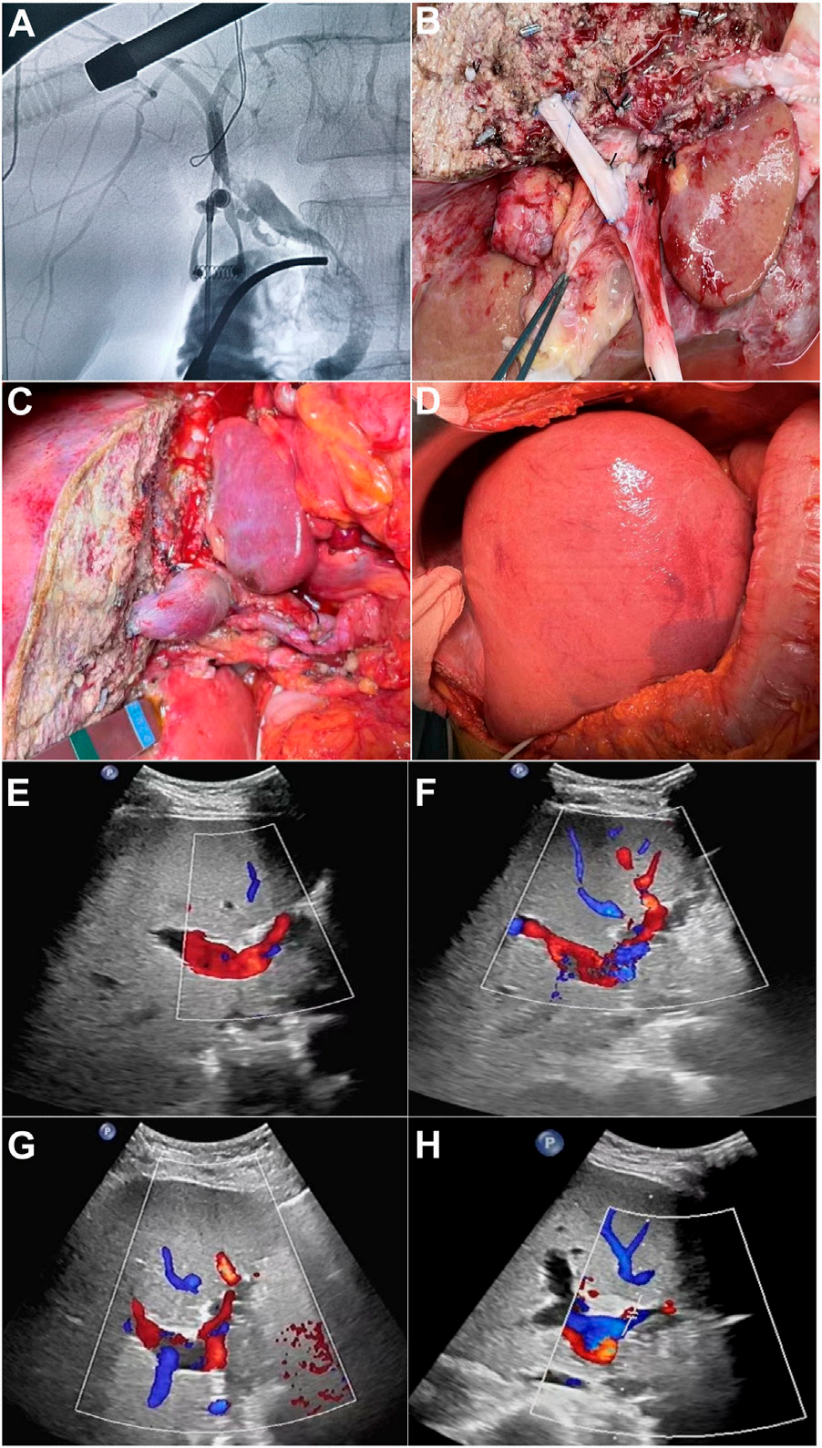
**(A)** Pre-splitting cholangiography of liver parenchyma. **(B)** Segment IV portal vein reconstruction before blood flow. **(C)** Segment IV portal vein reconstruction after blood flow. **(D)** After surgery/portal vein reconstruction, right three lobes. **(E–H)** Postoperative ultrasound images of segment IV portal vein bridging vessels of days 1, 3, 5, and 7, respectively.

This was a single center study, and after the necessary approval, the medical records of all patients who underwent split liver transplant were obtained. During the study from January 2015 to January 2022, a total of 32 cases of split liver transplant were obtained in which right trefoil hepatic portal vein reconstruction was carried out in 18 cases, and non-reconstruction of hepatic portal vein segment IV made up 14 cases and the general information of patients is shown in Table 1. In split liver transplantation, blood vessel splitting and distribution are the key to the success or failure of the operation. The choice of middle hepatic vein during left and right liver splitting is determined according to the situation of the two recipients before operation. Our center has made a series of improvements to the *invivo* split liver transplantation technology, especially the intraoperative vascular reconstruction. This mainly includes reconstruction of the segment IV portal vein after left lateral lobe and right trilobe splitting, left and right half liver splitting, reconstruction of middle hepatic vein after splitting, and formation of posterior vena cava and portal vein after splitting. The vascular materials required for reconstruction mainly come from donor iliac vessels and all of above discussed procedures are shown in Figures 1A–D.

**TABLE 1:** General information of control and reconstruction group.

	Control	Reconstruction	t/x2	*p*-value
Male	5	10	—	—
Female	9	8	—	—
Total	14	18	—	—
Age (Mean)Yrs	50.0 ± 12.48	50.66 ± 14.45	−0.667	0.8918
Height	162.57 ± 9.13	167.22 ± 7.90	0.1333	−4.651
Weight	57.28 ± 13.04	64.27 ± 9.55	−6.992*	0.0901
BMI	21.38 ± 3.25	22.93 ± 2.52	−1.548	0.1458
MELD Score (Points)†	16.42 ± 8.38	10.84 ± 6.62	5.584**	0.0475
Intraoperative conditions				
Weight of graft	1207.28 ± 267.69	1238.55 ± 198.14	31.270	0.7066
ALT/g	0.472 ± 0.276	0.413 ± 0.242	−0.059	0.5227
AST/g	0.714 ± 0.421	0.723 ± 0.354	0.009	0.9481
Operation time (min)	677.85 ± 122.65	603.611 ± 85.40	74.246*	0.0546
Anhepatic time (min)	52.21 ± 13.26	54.333 ± 16.60	−2.119	0.7071
Cold ischemia (min)	300.14 ± 21.46	302.50 ± 47.15	−2.357	0.8878
Blood transfusion RBC(U)	12.64 ± 5.57	9.05 ± 3.83	3.587*	0.0575
Postoperative recovery				
ICU (days)	4.78 ± 1.57	4.66 ± 1.64	0.119	0.8376
Postoperative hospital stay	36.28 ± 19.18	32.38 ± 7.88	3.897	0.4394

*** , ** and * shows the level of significance at 1%, 5% and 10%, respectively.

### Donor and Recipient Criteria

Recipients received right hemihepatic and right trilobular liver transplantation in our center if they met the following inclusion criteria: 1) Indications for liver transplantation with no contraindications; 2) preoperative Model for end-stage liver disease (MELD) score <30 points(17); 3) Graft weight/recipient weight (GRWR) ≥ 1.2% in adult recipients and 2%–4% in pediatric recipients (18); 4) No history of multiple abdominal surgeries and the donor also needed to have met the Milan recommendation criteria (19). The donor selection criteria included: 1) age <55 years; 2) hemodynamically stable, no need for high dose escalation maintenance with antidepressants (dopamine ≤5 mg/kg∙min, dobutamine ≤10 mg/kg∙min, no epinephrine or norepinephrine); 3) Intensive care unit (ICU) Hospitalization days <5 days; 4) aspartate transaminase (AST) and alanine aminotransferase (ALT) values lower than two times the normal value; 5) no fatty liver manifestations under the naked eye, if liver biopsy is performed, fatty infiltration moisture <20%; 6) Serum sodium <155 mmol/L. All donor livers which were cardiac-death organ donations were signed by immediate family members, and organ donation consent was given. All recipients signed the patient’s informed consent approved by the hospital ethics committee, in line with medical ethics regulations.

### Grouping and Observation Metrics

Of 32 patients, those who did not undergo portal vein reconstruction were included in the control group, consisting of 14 cases in total, and the 18 patients who underwent portal vein reconstruction were included in the reconstruction group. From the first to seventh days after the operation, ALT, AST, albumin (ALB), creatinine (Cr), total bilirubin (TB), alkaline phosphatase (ALP), gamma-glutamyl Transferase (GGT), lactic acid (Lac), and international normalized ratio (INR) level data were collected to analyze the significance of portal vein reconstruction of donor liver segment IV in patients with early postoperative liver function recovery.

### Statistical Methods

STATA statistical software was used for data processing, normally distributed measurement data were expressed as mean ± standard deviation, and *t*-test was used for independent samples. The reconstruction group was compared with the control group. Data with a *p*-value of less than 0.05 were considered statistically significant.

## Results

### Comparison of Postoperative Data of Recipients in the Reconstruction and Control Groups

There was a statistically significant difference between the control and reconstruction groups in the levels of ALT on days 2, 4, 5, 6, and 7 after surgery, and in the levels of Lac on day 1 after surgery, but there was no statistically significant difference in AST, ALB, Cr, TB, ALP, GGT, and INR as determined by a comparison of data which is shown in Table 2.

**TABLE 2:** Postoperative data comparison.

	Control	Value	Reconstruction	Value	t/x2	*p*-value
Postoperative ALT comparison (U/L)						
ALT1	14	942.50 ± 574.05	18	741.94 ± 235.13	200.556	0.1875
ALT2	14	759.42 ± 502.56	18	546.83 ± 149.02	212.595^*^	0.0980
ALT3	14	495.42 ± 292.19	18	374.88 ± 161.88	120.540	0.1478
ALT4	14	353.69 ± 196.94	18	240.88 ± 79.73	112.803**	0.0434
ALT5	14	231.92 ± 128.51	18	145.33 ± 73.50	86.595**	0.0226
ALT6	14	186.41 ± 101.71	18	106.16 ± 66.38	80.250**	0.0136
ALT7	14	117.46 ± 50.02	18	80.83 ± 55.75	36.628*	0.0785
Postoperative Lac comparison (mmol/L)						
Lac1	14	2.00 ± 1.36	18	3.17 ± 1.21	−1.163**	0.0194
Lac2	14	1.32 ± 0.55	18	1.40 ± 0.56	−0.071	0.7257
Lac3	14	1.26 ± 0.47	18	1.18 ± 0.59	0.078	0.6979
Lac4	14	1.26 ± 0.57	18	1.27 ± 0.55	−0.013	0.9513
Lac5	14	1.41 ± 0.76	18	1.31 ± 0.54	0.104	0.8152
Lac6	14	0.92 ± 0.36	18	1.28 ± 0.58	−0.358	0.3908
Lac7	14	1.40 ± 0.65	18	1.11 ± 0.71	0.286	0.4891
Postoperative AST comparison (U/L)						
AST1	14	1125.07 ± 1075.57	18	885.33 ± 405.62	239.738	0.3899
AST2	14	549.57 ± 374.29	18	443.33 ± 218.22	106.238	0.3221
AST3	14	241.42 ± 157.11	18	224.77 ± 128.40	16.651	0.7436
AST4	14	146.76 ± 93.67	18	126.33 ± 79.69	20.436	0.5206
AST5	14	100.28 ± 90.25	18	95.44 ± 84.26	4.841	0.8768
AST6	14	91.58 ± 80.57	18	69.16 ± 46.03	22.417	0.2820
AST7	14	71.69 ± 60.55	18	63.77 ± 34.10	7.915	0.6554
Postoperative ALB comparison (U/L)						
ALB1	14	40.22 ± 6.45	18	42.87 ± 6.09	−2.655	0.2616
ALB2	14	41.44 ± 4.59	18	41.22 ± 5.52	0.218	0.9091
ALB3	14	38.30 ± 8.16	18	39.27 ± 5.74	−0.972	0.7064
ALB4	14	38.83 ± 5.29	18	39.43 ± 5.82	−0.606	0.7842
ALB5	14	37.96 ± 6.40	18	40.11 ± 6.23	−2.142	0.3738
ALB6	14	35.49 ± 8.07	18	38.55 ± 5.28	−3.065	0.2211
ALB7	14	36.25 ± 8.72	18	37.16 ± 5.30	−0.907	0.7321
Postoperative ALP comparison (U/L)						
ALP1	14	90.54 ± 48.89	18	97.938 ± 53.24	−7.392	0.7308
ALP 2	14	143.09 ± 109.84	18	102.688 ± 57.20	40.403	0.3828
ALP 3	14	153.18 ± 133.19	18	101.063 ± 44.78	52.119	0.2289
ALP 4	14	177.30 ± 176.76	18	100.063 ± 39.37	77.238	0.1408
ALP 5	14	151.63 ± 121.61	18	100.267 ± 35.31	51.370	0.2310
ALP 6	14	138.55 ± 100.92	18	107.533 ± 44.02	31.022	0.3223
ALP 7	14	121.63 ± 70.58	18	110.333 ± 44.27	11.303	0.6507
Postoperative GGT comparison (U/L)						
GGT1	14	108.25 ± 138.24	18	53.063 ± 34.46	55.188	0.1655
GGT 2	14	84.18 ± 48.12	18	60.125 ± 34.69	24.057	0.1839
GGT 3	14	94.72 ± 48.91	18	65.563 ± 33.07	29.165	0.1263
GGT 4	14	114.50 ± 61.18	18	76.063 ± 37.58	38.438	0.1339
GGT 5	14	112.45 ± 50.36	18	87.933 ± 44.86	24.521	0.2637
GGT 6	14	113.33 ± 47.13	18	97.267 ± 49.50	16.067	0.4285
GGT 7	14	102.36 ± 36.74	18	106.733 ± 58.78	−4.370	0.8287
Postoperative Cr comparison (mmol/L)						
Cr1	14	93.91 ± 31.28	18	79.82 ± 27.80	14.082	0.1885
Cr2	14	80.55 ± 25.96	18	81.95 ± 26.62	−1.398	0.8826
Cr3	14	75.54 ± 22.73	18	71.16 ± 26.61	4.380	0.6266
Cr4	14	74.80 ± 15.50	18	65.99 ± 21.76	8.807	0.2102
Cr5	14	75.27 ± 25.70	18	62.44 ± 20.17	12.829	0.1238
Cr6	14	64.50 ± 17.63	18	63.66 ± 22.56	0.835	0.9198
Cr7	14	56.67 ± 19.03	18	61.05 ± 24.24	−4.381	0.6166
Postoperative TB comparison (µmol/L)						
TB1	14	76.20 ± 63.89	18	71.81 ± 35.09	4.390	0.8058
TB2	14	74.72 ± 48.48	18	70.76 ± 43.01	3.960	0.8086
TB3	14	65.38 ± 52.76	18	63.77 ± 40.03	1.619	0.9219
TB4	14	52.16 ± 37.59	18	73.14 ± 70.8	−20.978	0.3419
TB5	14	57.71 ± 37.32	18	62.53 ± 53.42	−4.815	0.7763
TB6	14	61.39 ± 37.96	18	52.81 ± 33.83	8.579	0.5393
TB7	14	57.25 ± 41.97	18	46.810 ± 25.21	10.440	0.4126
Postoperative INR comparison						
INR1	14	1.60 ± 0.39	18	1.53 ± 0.24	0.067	0.5587
INR 2	14	1.44 ± 0.24	18	1.40 ± 0.17	0.045	0.5509
INR 3	14	1.36 ± 0.24	18	1.29 ± 0.20	0.070	0.3877
INR 4	14	1.34 ± 0.43	18	1.30 ± 0.21	0.042	0.7248
INR 5	14	1.30 ± 0.34	18	1.27 ± 0.21	0.033	0.7394
INR 6	14	1.29 ± 0.32	18	1.25 ± 0.22	0.035	0.7176
INR 7	14	1.26 ± 0.29	18	1.24 ± 0.24	0.022	0.8169

*** , ** and * shows the level of significance at 1%, 5% and 10%, respectively.

### Significance of Portal Vein Reconstruction of Donor Liver Segment IV on Early Postoperative Liver Function Recovery

Judging from the recovery of various indicators of the recipients in the two groups after surgery, the recovery of liver function in the reconstruction group was significantly better than that in the control group. No serious bleeding, biliary fistula, or other complications occurred in the two groups of recipients after operation. In the control group, 14 recipients did not undergo segment IV hepatic portal vein reconstruction, and the segment IV liver was insufficiently perfused. Ultrasonography indicated that segment IV liver atrophy and necrosis occurred earlier, and early postoperative liver function recovery was poor. The postoperative ultrasound of the 18 recipients in the reconstruction group showed blood flow through the reconstructed vessels of the portal vein in the recipients within 1 week after surgery, the speed of IV segment liver atrophy was significantly slower than that of the control group, and the postoperative liver function recovered faster. It can be seen that intraoperative reconstruction of the portal vein of the donor liver segment IV can effectively reduce the damage of hepatocytes, preserve more functional liver tissue, and promote the early postoperative liver function recovery of patients, thereby improving the prognosis of patients and restoring blood recirculation, which is shown in Figures 1E–H.

### Survival Analysis

We compared the 6-month data to calculate the survival rate between these two groups.

**FIGURE 2:**
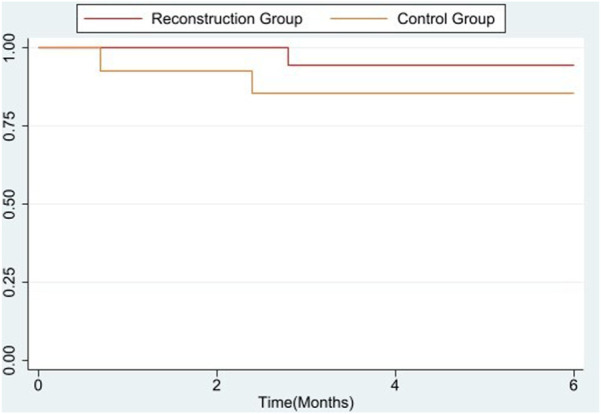
Survival rate analysis of 6 months between control and reconstruction group.

The 6-month rate in the Control group and Reconstruction group was [85.7% vs. 94.4%] with an overall 90.6% rate of survival. There was no significant difference in survival rate between these two groups is shown in the Figure 2.

## Discussion

The development of split liver transplant was prompted by a lack of organs and rising morbidity on waiting lists. The gap between organ supply and recipient demand has never been wider than it is today. This has rekindled interest in expanding the use of traditional adult/pediatric split liver transplant and adult/adult split liver transplant. At centers that routinely use these techniques, split liver transplant applied to pediatric recipients offers good results, with considerable decreases in pediatric wait times, wait-list morbidity, and living-donation utilization, according to a decade of experience with left lateral segment grafts (20, 21). Split liver transplantation, as the most difficult liver transplantation technology, comprehensively embodies this feature from the preoperative evaluation of the general conditions of donors and recipients, to the distribution of blood vessels including hepatic artery, portal vein, inferior vena cava, and hepatic vein and biliary tract. The splitting of liver parenchyma, the acquisition of donor liver, and the fine individual management after operation represents the forefront of the development of precision medicine (22). With the accumulation of clinical experience, especially the deepening of the research on the local anatomical structure of the liver, and the continuous summary and exchange of the experience of multi center split liver transplantation, the effect of split liver transplantation has been significantly improved (23).

The recipient selection of donation after brainstem death (DBD) orthotopic split liver transplantation is also key to the success of the transplantation, especially when the recipient is a double adult split liver transplantation. The matching degree between the graft size and the recipient needs to be carefully evaluated before operation to prevent the possibility of small for size syndrome or large for size syndrome due to the mismatch between the donor and the recipient. The GRWR standard of DBD orthotopic split liver transplantation should be appropriately increased compared with living donor liver transplantation, and it is recommended to be greater than 1.0%–1.2% (24). The GRWR of adult recipients of DBD *in situ* split liver transplantation in our center was controlled at more than 1.0%, and there was no obvious small liver syndrome after operation. We believe that the hyperoxia environment of the transplanted liver is beneficial to the regeneration of liver cells.

Therefore, the receptor should be emphasized to ensure long-term oxygen inhalation after operation. CT examination involves radiation and may affect the regeneration of liver cells. Therefore, we suggest that abdominal CT examination should be avoided as much as possible in the early stage after operation. Split liver transplantation is complex, the operation time is relatively increased, there are risks such as cross-sectional bile leakage and infection after operation, and the general requirements for the recipient are high because relevant studies show that a high MELD score before operation is an independent risk factor for serious complications after liver transplantation, so care should be taken to select recipients with a MELD score >14 for split liver transplantation (17, 18, 25). The donor liver splitting operation in our center adopts *in situ* splitting *in vivo* compared with the traditional *in vitro* splitting after acquisition, it can significantly reduce the cold ischemia time, dissect the hilar tissue more finely, deal with the liver section more accurately, and reduce the incidence of postoperative complications. It is suggested that the middle hepatic vein should be accurately located by intraoperative ultrasound before splitting, and perfusion can be carried out after splitting when the middle hepatic vein is exposed (26).

Although the vascular reconstruction and repair molding of the left and right liver halves respectively increase the operation time, it ensures that the left and right liver grafts have a complete middle hepatic vein system, the operation method is more reasonable, the necrosis of the graft liver tissue caused by outflow tract obstruction is avoided, and the functional liver volume of the graft is effectively increased. After the blood supply of the donor liver is restored during the operation, the sections of each anastomosis and liver parenchyma are comprehensively checked, and the bleeding points and broken ends with bile leakage are also treated in time. The reconstruction of the artery is flexibly evaluated according to the distribution of the donor hepatic artery and the recipient’s own arterial conditions. If the length of the vessel is not sufficient, the donor iliac vessel can be used for bridging if necessary. In general, T-tube drainage is routinely placed in our center. The advantages of T-tube drainage are as follows: first, the recovery of donor liver function and the occurrence of rejection can be evaluated by observing the amount and color of drained bile in the early stage after operation; The second is that it can fulfil the role of biliary decompression before the recovery of gastrointestinal function, so as to reduce the occurrence of biliary complications such as bile leakage.

In conclusion, split liver transplantation can effectively alleviate the problem of liver shortages. Split liver transplantation can give the same clinical results as whole liver transplantation if the right donors and recipients are chosen and if the surgery is planned and carried out well. With the continuous maturity and progress of split liver transplantation technology, split liver transplantation is expected to become a routine operation in clinical liver transplantation and to become widely used. To sum up, our center’s exploration of portal vein reconstruction in segment IV of the liver in split liver transplantation is a reasonable attempt based on anatomy, which is conducive to the recovery of patient’s early postoperative liver function in clinical practice. However, because this surgical method is in the early exploratory stage, the number of samples included in this study is limited and, due to the differences in the technical level of the operators, the results have certain limitations that need to be further verified by continuing to expand the sample size in a multi-center practice.

## Data Availability

The raw data supporting the conclusion of this article will be made available by the authors, without undue reservation.

## Abbreviations

ALB, albumin; ALP, alkaline phosphatase; ALT, alanine aminotransferase; AST, aspartate transaminase; BMI, body mass index; Cr, creatinine; DBD, donation after brainstem death; GGT, gamma-glutamyl transferase; GRWR, graft weight/recipient weight; ICU, intensive care unit; INR, international normalized ratio; Lac, lactic acid; MELD, model for end-stage liver disease; TB, total bilirubin.
